# Induction of NLRP3 Inflammasome Activation by Heme in Human Endothelial Cells

**DOI:** 10.1155/2018/4310816

**Published:** 2018-03-20

**Authors:** Judit Erdei, Andrea Tóth, Enikő Balogh, Benard Bogonko Nyakundi, Emese Bányai, Bernhard Ryffel, György Paragh, Mario D. Cordero, Viktória Jeney

**Affiliations:** ^1^Department of Internal Medicine, Faculty of Medicine, University of Debrecen, Debrecen 4032, Hungary; ^2^The National Center for Scientific Research (CNRS), UMR7355, Experimental and Molecular Immunology and Neurogenetics, 45071 Orleans, France; ^3^Institute of Infectious Disease & Molecular Medicine (IDM), University of Cape Town, Cape Town, South Africa; ^4^Department of Physiology, Institute of Nutrition and Food Technology “José Mataix Verdú”, Biomedical Research Center, University of Granada, 18100 Granada, Spain

## Abstract

Hemolytic or hemorrhagic episodes are often associated with inflammation even when infectious agents are absent suggesting that red blood cells (RBCs) release damage-associated molecular patterns (DAMPs). DAMPs activate immune and nonimmune cells through pattern recognition receptors. Heme, released from RBCs, is a DAMP and induces IL-1*β* production through the activation of the nucleotide-binding domain and leucine-rich repeat-containing family and pyrin domain containing 3 (NLRP3) in macrophages; however, other cellular targets of heme-mediated inflammasome activation were not investigated. Because of their location, endothelial cells can be largely exposed to RBC-derived DAMPs; therefore, we investigated whether heme and other hemoglobin- (Hb-) derived species induce NLRP3 inflammasome activation in these cells. We found that heme upregulated NLRP3 expression and induced active IL-1*β* production in human umbilical vein endothelial cells (HUVECs). LPS priming largely amplified the heme-mediated production of IL-1*β*. Heme administration into C57BL/6 mice induced caspase-1 activation and cleavage of IL-1*β* which was not observed in NLRP3^−/−^ mice. Unfettered production of reactive oxygen species played a critical role in heme-mediated NLRP3 activation. Activation of NLRP3 by heme required structural integrity of the heme molecule, as neither protoporphyrin IX nor iron-induced IL-1*β* production. Neither naive nor oxidized forms of Hb were able to induce IL-1*β* production in HUVECs. Our results identified endothelial cells as a target of heme-mediated NLRP3 activation that can contribute to the inflammation triggered by sterile hemolysis. Thus, understanding the characteristics and cellular counterparts of RBC-derived DAMPs might allow us to identify new therapeutic targets for hemolytic diseases.

## 1. Introduction

Damage-associated molecular patterns (DAMPs) or alarmins are endogenous biomolecules that are released upon tissue stress, injury, or cell death. DAMPs are able to trigger and/or exacerbate innate immune response via the activation of diverse innate immune receptors [1]. Hemolytic or hemorrhagic episodes are often associated with inflammation even when infectious agents are absent, suggesting that damaged red blood cells (RBCs) release DAMPs [2, 3].

The far most abundant protein in mature RBCs is hemoglobin (Hb) that composes 96% of the dry weight of RBCs; therefore, upon hemolysis, tremendous amounts of Hb are released into the extracellular milieu. Outside of the protective environment of RBCs, Hb is prone to oxidation, leading to the formation of oxidized Hb forms, that is, metHb (MHb) and ferrylHb (FHb) [4–10]. Because of conformational changes, oxidized Hb forms release their heme prosthetic group. An endogenous protective system evolved to limit the harmful effects of extracellular Hb and heme that relies mainly on the presence of two proteins in the plasma, namely, haptoglobin and hemopexin. These acute-phase proteins scavenge Hb and heme, respectively, and help their efficient removal from the circulation [11–14]. Upon massive intravascular hemolysis, this protective system becomes overwhelmed, leading to the depletion of haptoglobin and hemopexin and the accumulation of Hb and heme in the plasma [11–14].

Extracellular Hb, particularly in its oxidized forms and the released heme, exerts various biological effects. Heme is a potent prooxidant and proinflammatory molecule (reviewed in [10, 15]). As a prooxidant, heme induces lipid peroxidation and sensitizes various cell types to oxidant- and tumor necrosis factor- (TNF-) mediated programmed cell death [16–19]. As a proinflammatory agonist, heme targets macrophages and induces TNF secretion via a toll-like receptor 4- (TLR4-) dependent mechanism [20] and triggers interleukin 1 beta (IL-1*β*) production through a mechanism dependent on the expression of the nucleotide-binding domain and leucine-rich repeat-containing protein 3 (NLRP3) inflammasome [21]. Through heme release, metHb and ferrylHb share most of the deleterious effects of free heme [10, 19, 22].

Endothelial cells provide a barrier between blood and tissue and therefore play a fundamental role in the inflammatory response. Because of their location, endothelial cells are in the frontline to be exposed to Hb and its oxidation products upon intravascular hemolysis [23]. Growing evidence suggests that heme and oxidized Hb species play a pathophysiological role in endothelial cell activation upon hemolytic diseases via the upregulation of adhesion molecules [24–26]. Additionally, endothelial cells respond to different alarmins by NLRP3 inflammasome activation and subsequent release of IL-1*β*, and this mechanism has been shown to play a significant role in diverse pathological conditions including atherosclerosis, diabetic retinopathy, diabetic nephropathy, and chronic kidney disease [27–32].

Heme is a prototypical alarmin that triggers NLRP3 activation in macrophages, but we lack knowledge on whether it acts on endothelial cells. Therefore, here, we investigated whether heme or other Hb-associated DAMPs induce NLRP3 inflammasome activation in human umbilical vein endothelial cells (HUVECs).

## 2. Materials and Methods

### 2.1. Materials

Reagents were purchased from Sigma-Aldrich (St. Louis, MO, USA) unless otherwise specified.

### 2.2. Mice

C57BL/6 and *Nlrp3^−/−^* mice were maintained at the University of Debrecen in a conventional animal house and were used between 6 and 8 weeks of age. All experiments followed guidelines of the institutional and national ethical committee and underwent approval. The *Nlrp3^−/−^* mice strain was originally generated and characterized in the laboratory of J. Tschopp [33]. To study the inflammatory action of heme, twenty C57BL/6 mice (female, 6–8 weeks of age) were randomly divided into 4 groups (*n* = 5/group) and injected intraperitoneally (i.p.) with heme at a dose of 75, 150, and 300 nmol/peritoneal cavity in 200 *μ*L apyrogen PBS. Control mice received PBS only. In one experiment, we injected C57BL/6 mice (*n* = 4) i.p. with heme-albumin that was prepared by incubating heme with an equimolar amount of human albumin for 10 minutes at room temperature. After 16 hours, mice were sacrificed by CO_2_ exposure and peritoneal leukocytes were harvested by peritoneal lavage using ice-cold PBS containing 2% FCS (Gibco, Thermo Fisher Scientific Inc., Waltham, MA, USA) and were analyzed by flow cytometry. Total number of cells was determined using a fixed number of latex beads (Beckman Coulter, Paris, France), coacquired with a preestablished volume of the cell suspensions. Number of peritoneal neutrophils was evaluated using R-phycoerythrin- (R-PE-) conjugated rat anti-mouse Ly-6G (Gr1; CD11b, BD Biosciences, San Jose, CA, USA) and biotin anti-mouse neutrophil monoclonal antibody (CL8993B, Cedarlane, Hornby, Ontario, Canada). Cells were costained with propidium iodide (0.5 *μ*g/mL) to exclude dead cells. Fluorescence was measured by flow cytometry (FACS Calibur, BD Biosciences), and data was analyzed using FlowJo software (Tree Star, Inc., Ashland, OR, USA). Ly-6G and 7/4 double positive cells were identified as neutrophils, Ly-6G negative and 7/4 positive cells were considered as inflammatory monocytes/macrophages [34]. In another experiment, twenty C57BL/6 mice (female, 6–8 weeks of age) were randomly divided into 4 groups (*n* = 5/group) and injected intraperitoneally (i.p.) with LPS (100 *μ*g/peritoneal cavity), heme (300 nmol/peritoneal cavity), or LPS + heme in 200 *μ*L apyrogen PBS. Control mice received PBS only. Total leukocytes in the peritoneal fluid was determined on Burker chambers after dilution in Turk solution as it was described previously [21]. For IL-1*β* detection, peritoneal fluid was centrifuged and the amount of IL-1*β* in the supernatants was quantified by ELISA (DuoSet ELISA, R&D, Minneapolis, MN, USA). To assess the role of NLRP3 in heme-mediated inflammatory response, 6 C57BL/6 and 6 NLRP3^−/−^ mice (female, 6–8 weeks of age) were randomly divided into 2 groups (*n* = 3/group) and injected i.p. with heme (300 nmol/cavity in 200 *μ*L PBS) or vehicle. Livers were collected 16 hours postinjection, frozen in liquid nitrogen, and stored at −70°C until analysis.

### 2.3. Cell Culture

Human umbilical vein endothelial cells (HUVECs) were removed from human umbilical cords (*n* = 8) by exposure to dispase and cultured in medium 199 containing 15% FBS, antibiotics, heparin, L-glutamine, sodium pyruvate, and endothelial cell growth factor on gelatinized plates as described previously [19].

### 2.4. Hemoglobin Preparation

Hb of different redox states, that is, oxyHb (Fe^2+^), metHb (Fe^3+^), and ferrylHb (Fe^4+^ = O), was prepared as described [25]. Briefly, Hb was isolated from fresh blood drawn from healthy volunteers using ion-exchange chromatography on a DEAE Sepharose CL-6B column. MetHb was generated by incubation (30 min, 25°C) of purified Hb with a 1.5-fold molar excess of K_3_Fe (CN)_6_ over heme. FerrylHb was obtained by incubation (1 h, 37°C) of Hb with a 10 : 1 ratio of H_2_O_2_ to heme. After oxidation, both metHb and ferrylHb were dialyzed against saline (3 times for 3 hours at 4°C) and concentrated using Amicon Ultra centrifugal filter tubes (10,000 MWCO, Millipore Corp., Billerica, MA, USA). Aliquots were snap-frozen in liquid nitrogen and stored at −70°C until use. Purity of each Hb preparation was evaluated by SDS-PAGE followed by staining with ProteoSilver Plus Silver Staining Kit. The purity of Hb preparations was above 99.9%. Hb concentrations were calculated as described by Winterbourn [4].

### 2.5. HUVEC Treatment

HUVECs were used at passage 2 and 3 within 2 days postconfluence. When indicated, HUVECs were pretreated with the indicated doses of lipopolysaccharide (LPS; 0.1, 1, and 10 *μ*g/mL in complete (15% FBS) medium) for 24 hours. Heme was dissolved in NaOH (20 mmol/L), the pH was adjusted slowly to 7.4 with HCl and the solution was sterile filtered using a 0.2 *μ*m syringe filter (Millipore). Heme treatments were carried out in 1% FBS-containing medium for 4 hours (mRNA), 12 hours (protein expression), or 24 hours (IL-1*β* secretion and viability assays).

### 2.6. Quantitative Real-Time PCR (qRT-PCR)

RNA was isolated from cells using TRIzol (RNA-STAT60, Tel-Test Inc., Friendswood, TX, USA) according to the manufacturer's protocol. Two micrograms of RNA were reverse transcribed to cDNA with High-Capacity cDNA Reverse Transcription Kit (Applied Biosystems, Waltham, MA, USA). Quantitative real-time PCR was performed using iTaq Universal Probes Supermix (Bio-Rad Laboratories, Hercules, CA, USA) and predesigned primers and probes (TaqMan® Gene Expression Assays) to detect *IL-1β* (Hs.00174097), *NLRP3* (Hs.00918082), *ASC* (Hs.01547324), *HO-1* (Hs.01110250), and *GAPDH* (Hs.02758991). Relative mRNA expressions were calculated with the ΔΔCt method using *GAPDH* as an internal control.

### 2.7. Determination of Cell Viability

Cell viability was determined by the MTT assay as previously described [19]. Briefly, following treatments, cells were washed with PBS, and 100 *μ*L of 3-[4,5-Dimethylthiazol-2-yl]-2,5-diphenyl-tetrazolium bromide (0.5 mg/mL) solution in HBSS was added. After a 4-hour incubation, the MTT solution was removed, formazan crystals were dissolved in 100 *μ*L of DMSO, and optical density was measured at 570 nm.

### 2.8. IL-1*β* Secretion in HUVECs

HUVECs were cultured in 96-well plates. Following treatment, cellular supernatants were collected and 100 *μ*L of undiluted sample was used for ELISA analysis (DuoSet ELISA, R&D, Minneapolis, MN, USA). All of the measurements were performed according to the manufacturer's protocol.

### 2.9. Western Blot

Following treatment, HUVECs were solubilized in 10 mmol/L TrisHCl, containing 5 mmol/L EDTA, 150 mmol/L NaCl (pH 7.2), 1% Triton X-100, 0.5% Nonidet P-40, and protease inhibitors (Complete Mini, F. Hoffmann-La Roche Ltd., Basel, Switzerland). Whole cell lysates (20 *μ*g) were used to evaluate NLRP3, HO-1, and GAPDH protein expressions. Liver lysates (20 *μ*g) obtained from C57BL/6 or *Nlrp3^−/−^* mice were used to investigate caspase-1 and IL-1*β* processing. Protein samples were run on 12.5% SDS-PAGE. Western blotting was performed with the use of a monoclonal anti-NLRP3 antibody (Clone number 768319, R&D Systems, Minneapolis, USA), a polyclonal anti-caspase-1 p20 antibody (sc-398,715, Santa Cruz Biotechnology Inc., Dallas, TX, USA), a monoclonal anti-IL-1*β* antibody (number 12242, Cell Signaling Technology, Leiden, Netherlands), and a monoclonal anti-human HO-1 antibody (sc-136,960, Santa Cruz) followed by the species-specific HRP-labeled secondary antibodies (Amersham Biosciences Corp., Piscataway, NJ, USA). Antigen-antibody complexes were visualized with the horseradish peroxidase chemiluminescence system (Amersham Biosciences Corp., Piscataway, NJ, USA). After detection, the membranes were stripped and reprobed for GAPDH using anti-GAPDH antibody at a dilution of 1 : 1000 (Novus Biologicals, Littleton, CO, USA). Results were quantified by using the Alpha DigiDoc RT (Alpha Innotech, San Leandro, CA, USA) quantification system.

### 2.10. Intracellular ROS Measurement

ROS production was monitored by using the 5-(and-6)-chloromethyl-2′,7′-dichlorodihydro-fluorescein di-acetate and acetyl ester (CM-H_2_DCFDA) assay (Life Technologies, Carlsbad, CA, USA). After the treatment, cells were washed with PBS and loaded with CM-H_2_DCFDA (10 *μ*mol/L, 30 min, in the dark). Cells were washed thoroughly, and fluorescence intensity was measured applying 488 nm excitation and 533 nm emission wavelengths for 3 hours in every 30 minutes. In some experiments, ROS was scavenged by N-acetyl cysteine (NAC, 5 mmol/L) during the treatments.

### 2.11. Statistical Analysis

Data are shown as mean ± S.D. Statistical analysis was performed by one-way ANOVA or Student's *t*-test, as appropriate. *P* < 0.05 was considered significant.

## 3. Results

### 3.1. Heme Acts as a Proinflammatory Agonist and Induces IL-1*β* Secretion In Vivo

**Figure 1:**
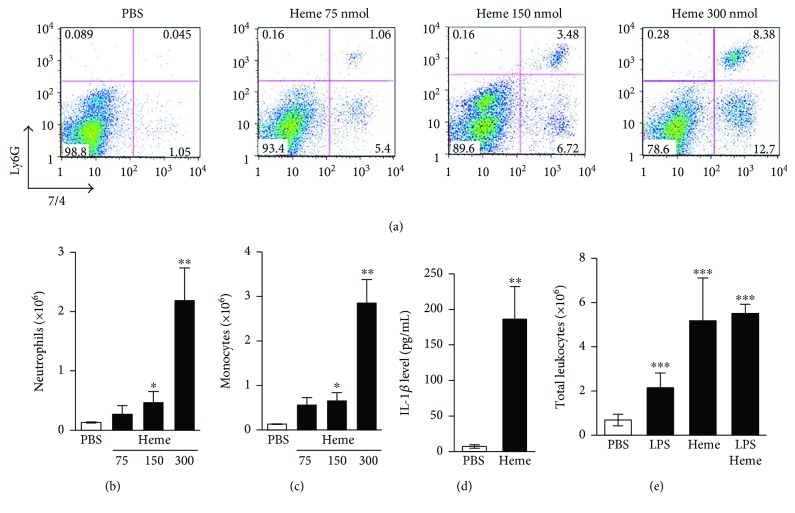
Heme induces neutrophil and monocyte infiltration and IL-1*β* secretion *in vivo*. (a–c) C57BL/6 mice were injected (i.p.) with heme (75, 150, or 300 nmol/mice) or PBS (*n* = 5 in all groups). Peritoneal cavity was rinsed, and neutrophil and inflammatory monocytes/macrophage numbers were determined after 16 h of treatment. (a) Representative dot plots of peritoneal cells stained with Ly-6G and 7/4. (b) Mean number of Ly-6G high and 7/4 high PMN cells. (c) Mean number of Ly-6G low and 7/4 high inflammatory monocytes/macrophages. (d) C57BL/6 mice were injected (i.p.) with heme (300 mmol/kg body weight) or vehicle. Peritoneal cavity was rinsed, and the active IL-1*β* level was determined in the supernatant by ELISA (*n* = 5 in both groups). (e) C57BL/6 mice were injected (i.p.) with LPS (100 *μ*g/mice), heme (300 nmol/mice), and LPS + heme or PBS (*n* = 5 in all groups). Peritoneal cavity was rinsed, and total numbers of leukocytes was counted after 16 h of treatment. Data represent mean ± SD; ^∗^*P* < 0.05, ^∗∗^*P* < 0.01, and ^∗∗∗^*P* < 0.005.

To examine whether heme exerts proinflammatory effects *in vivo*, we injected heme into the peritoneal cavity of C57BL/6 mice. Heme induced a dose-dependent inflammatory response, and its highest dose (300 nmol/mice) triggered about a 20-fold increase in the number of peritoneal PMN cells and monocytes/macrophages, as compared with vehicle-treated controls (Figures 1(a)–1(c)). Next, we analyzed the effect of heme on the production of IL-1*β in vivo*. Intraperitoneal administration of heme caused an about 25-fold increase in the peritoneal IL-1*β* level as compared with vehicle-treated controls (Figure 1(d)). These results indicate that heme triggers processing and release of IL-1*β in vivo*. We compared the proinflammatory effect of heme to that of LPS and found that heme at the dose of 300 nmol/mice triggered 2.9-fold more leukocyte infiltration into the peritoneal cavity than LPS (100 *μ*g/mice) (Figure 1(e)). LPS failed to further increase the number of infiltrating leukocytes triggered by heme (Figure 1(e)).

### 3.2. Heme Induces IL-1*β* Secretion in HUVECs through NLRP3 Inflammasome Activation

**Figure 2:**
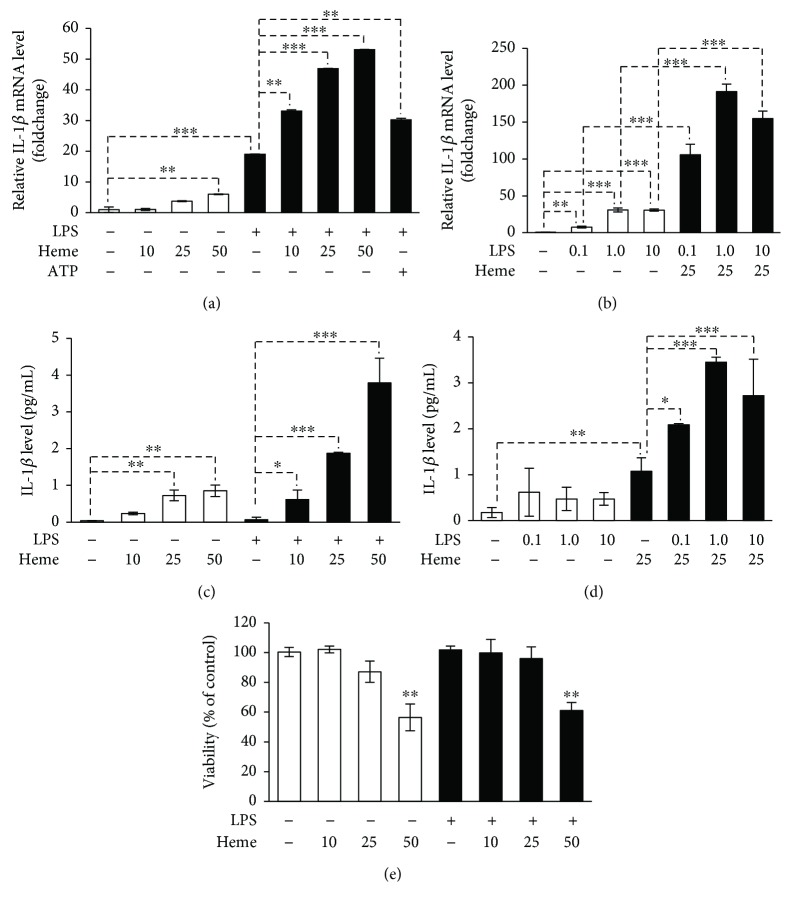
Heme induces IL-1*β* maturation and secretion in HUVECs. (a) LPS-primed (10 *μ*g/mL, 24 h) or nonprimed HUVECs were exposed to heme (10, 25, and 50 *μ*mol/L in 1% FBS, 4 h) or ATP (5 mmol/L). IL-1*β* mRNA levels were determined by qRT-PCR. (b) LPS-primed (0.1, 1, and 10 *μ*g/mL, 24 h) or nonprimed HUVECs were exposed to heme (25 *μ*mol/L in 1% FBS, 4 h). IL-1*β* mRNA levels were determined by qRT-PCR. (c) LPS-primed (10 *μ*g/mL, 24 h) or nonprimed HUVECs were exposed to heme (10, 25, and 50 *μ*mol/L in 1% FBS, 24 h). Secreted IL-1*β* levels were determined by ELISA from the cellular supernatant. (d) LPS-primed (0.1, 1, and 10 *μ*g/mL, 24 h) or nonprimed HUVECs were exposed to heme (25 *μ*mol/L in 1% FBS, 24 h). Secreted IL-1*β* levels were determined by ELISA from the cellular supernatant. (e) Cells were treated as in (c), and cellular viability was assessed by MTT assay. Results are shown as mean ± SD (*n* = 3) from one representative experiment of three. ^∗^*P* < 0.05, ^∗∗^*P* < 0.01, and ^∗∗∗^*P* < 0.005.

Dutra et al. showed that heme triggers IL-1*β* production in LPS-primed macrophages through the activation of the NLRP3/ASC/caspase-1 inflammasome platform [21]. Endothelial cells provide a barrier between blood and tissues, and therefore they are heavily exposed to heme upon intravascular hemolysis. Given this fact, we asked whether heme-induced inflammasome activation—besides of macrophages—also occurs in endothelial cells. To test this, we treated HUVECs with heme and measured IL-1*β* mRNA levels. We found that heme (50 *μ*mol/L) increased IL-1*β* mRNA levels by 6-fold compared to vehicle-treated cells (Figure 2(a)). In general, NLRP3 inflammasome activation requires two distinct signals; therefore, we next examined how heme behaves as a second signal. In this case, we pretreated HUVECs with LPS (signal 1) before the heme (signal 2) exposure. LPS treatment alone caused an about 19-fold increase in IL-1*β* mRNA levels as compared to vehicle control (Figure 2(a)). Furthermore, we found that LPS priming largely enhanced the heme response and this combined treatment resulted in a 53-fold elevation in IL-1*β* mRNA (Figure 2(a)). We checked whether IL-1*β* mRNA response is dependent on the dose of LPS. We primed HUVECs with different doses of LPS and found that LPS at the dose of 0.1 *μ*g/mL efficiently amplified the effect of heme on IL-1*β* mRNA levels (Figure 2(b)). Next, we asked whether heme triggers processing and secretion of active IL-1*β*. For this, we measured the level of processed IL-1*β* in the cellular supernatant of HUVECs. We found that heme alone caused a dose-dependent mild increase in the level of active IL-1*β* as compared to vehicle-treated cells. LPS treatment did not increase secreted IL-1*β* levels but largely enhanced the heme-mediated response (Figures 2(c) and 2(d)). To see whether cell death is involved in inflammasome activation by heme, we assessed cellular viability following the treatments. We found that heme up to 25 *μ*mol/L is not toxic, but we observed an about 40% of cell death when cells were exposed to 50 *μ*mol/L heme (Figure 2(e)). The effect of heme on cell viability was independent of LPS priming (Figure 2(e)). These results suggest that heme and LPS act synergistically to induce the secretion of IL-1*β* in endothelial cells.

**Figure 3:**
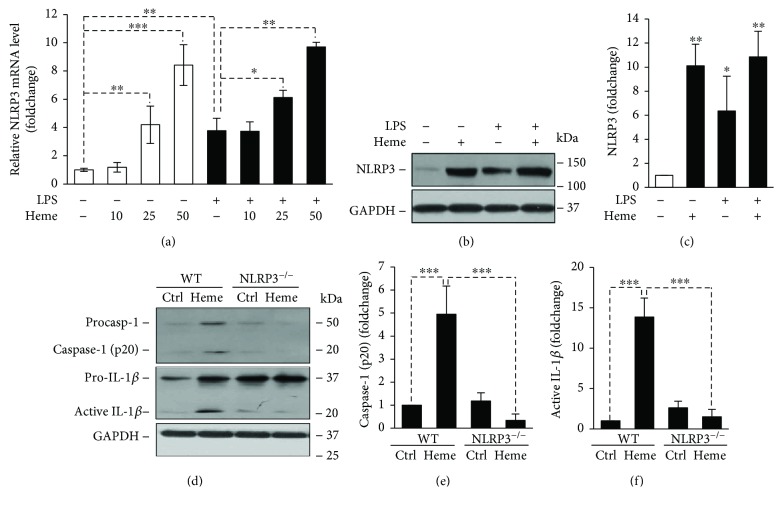
Heme induces NLRP3 expression and activation of caspase-1. (a) LPS-primed (10 *μ*g/mL, 24 h) or nonprimed HUVECs were exposed to heme (10, 25, and 50 *μ*mol/L in 1% FBS, 4 h). NLRP3 mRNA levels were determined by qRT-PCR. (b) LPS-primed (10 *μ*g/mL, 24 h) or nonprimed HUVECs were exposed to heme (25 *μ*mol/L in 1% FBS, 6 h). NLRP3 were analyzed by Western blot from whole cell lysate. Membrane was reprobed for GAPDH. Representative blots of 2 independent experiments are shown. (c) Densitometric analysis of Western blots. (d) C57BL/6 and *Nlrp3^−/−^* mice were injected with heme (300 nmol/peritoneal cavity) or vehicle (Ctrl). Protein expressions of activated caspase-1 and processed IL-1*β* were analyzed by Western blot from liver samples (16 h). Membrane was reprobed for GAPDH. Representative blots of 3 independent experiments are shown. (e and f) Densitometric analysis of Western blots. Results are shown as mean ± SD of 3 independent experiments. ^∗^*P* < 0.05, ^∗∗^*P* < 0.01, and ^∗∗∗^*P* < 0.005.

To see whether NLRP3 inflammasome activation is involved in the heme-mediated production of IL-1*β,* first, we checked mRNA and protein levels of NLRP3. We found that heme increased NLRP3 mRNA and protein expressions in HUVECs (Figures 3(a)–3(c)). LPS as well caused elevation of NLRP3 expression on both mRNA and protein levels but failed to further increase the heme-mediated responses (Figures 3(a)–3(c)). Assembly of the NLRP3 inflammasome platform results in activation of caspase-1. We assessed whether heme treatment triggers caspase-1 activation *in vivo*. Injection of heme into wild-type mice peritoneum induced an almost five-fold elevation in the level of active caspase-1 (p20) in the liver (Figures 3(d) and 3(e)). In contrast, no heme-mediated caspase-1 activation occurred in *Nlrp3^−/−^* mice (Figures 3(d) and 3(e)). Heme induced a 13.8-fold increase in the level of processed active IL-1*β* in the liver of wild-type mice. On the contrary, we did not observe active IL-1*β* formation in heme-treated *Nlrp3^−/−^* mice (Figures 3(d) and 3(f)). These results suggest that heme-mediated production of IL-1*β* occurs through the activation of NLRP3 inflammasome and caspase-1 activation.

### 3.3. Coordinated Iron of the Porphyrin Ring Is Involved in Heme-Mediated Inflammasome Activation

**Figure 4:**
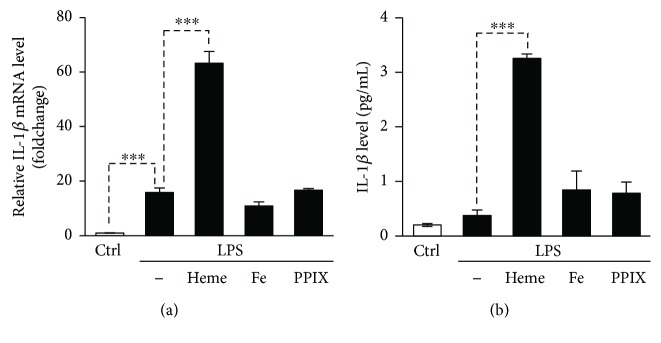
The coordinated iron is critical in heme-mediated induction of IL-1*β*. (a and b) HUVECs primed with LPS (10 *μ*g/mL, 24 h) were stimulated with heme, PPIX, or FeSO_4_ (25 *μ*mol/L). (a) IL-1*β* mRNA level (4 h) was determined by quantitative RT-PCR. (b) Active IL-1*β* levels in cellular supernatants (24 h) were determined by ELISA. Results are shown as mean ± SD (*n* = 3) from one representative experiment of three. ^∗∗∗^*P* < 0.005.

Next, we aimed to explore the structural motifs that are involved in heme-mediated inflammasome activation. We tested protoporphyrin IX (PPIX), a precursor of heme that lacks the central Fe^2+^ ion, and the iron salt FeSO_4_ on whether they are able to trigger the production of IL-1*β* in HUVECs. Both PPIX and free iron (Fe^2+^) failed to increase the level of IL-1*β* mRNA in LPS-pretreated HUVECs (Figure 4(a)). Stimulation of LPS-treated HUVECs with PPIX or free iron did not cause secretion of mature IL-1*β* (Figure 4(b)). These results suggest that coordinated iron present in the heme molecule, but not the protoporphyrin ring or the released iron, is critical to the activation of the inflammasome.

### 3.4. ROS Are Involved in Heme-Mediated Inflammasome Activation

**Figure 5:**
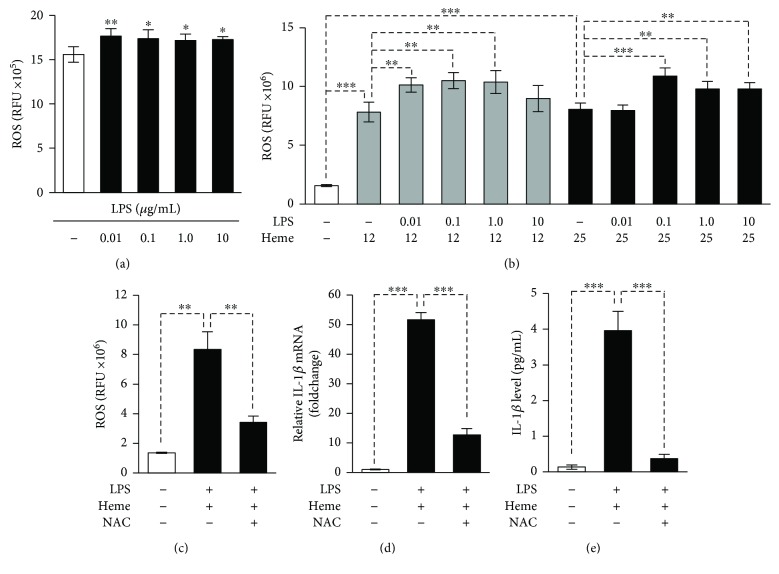
Unfettered ROS production is critical in heme-mediated induction of IL-1*β*. (a) HUVECs were treated with LPS (0.01–10 *μ*g/mL, 24 h). (b) HUVECs were primed with LPS (0.01–10 *μ*g/mL, 24 h) then treated with heme (12.5 or 25 *μ*mol/L, 4 h). (c–e) Naive or LPS-primed (10 *μ*g/mL, 24 h) HUVECs were treated with heme (25 *μ*mol/L) in the presence or absence of NAC (5 mmol/L). (a–c) Following the 4-hour heme treatment, ROS production was measured with DCFDA assay. (d) Following the 4-hour heme treatment, IL-1*β* mRNA level was determined by quantitative RT-PCR. (e) Active IL-1*β* levels in cellular supernatants (24 h) were determined by ELISA. Results are shown as mean ± SD (*n* = 5) from one representative experiment of three. ^∗∗∗^*P* < 0.005, ^∗∗^*P* < 0.01, and ^∗^*P* < 0.05.

Both heme and LPS are well-known inducers of ROS production in endothelial cells [18, 35]. Recent studies highlighted the critical involvement of ROS in NLRP3 activation induced by several stimuli [36, 37]; therefore, we next examined whether elevated ROS production plays a role in heme-mediated inflammasome activation in endothelial cells. First, we investigated ROS production triggered by different doses of LPS in HUVECs. We found that LPS—at the concentration range from 100 ng/mL to 10 *μ*g/mL—slightly but significantly increased ROS production in HUVECs (Figure 5(a)). Then, we investigated whether these doses of LPS could increase ROS production triggered by heme. Our results revealed that heme is a very potent inducer of ROS production in HUVECs, resulting in about 5-fold elevation of ROS production over controls and that LPS priming slightly but significantly increased heme-triggered ROS formation (Figure 5(b)). The radical scavenger NAC partially inhibited ROS formation in LPS-primed heme-treated HUVECs (Figure 5(c)). This was associated with the reduction of heme-induced upregulation of IL-1*β* mRNA and the attenuation of active IL-1*β* formation in LPS-primed endothelial cells (Figures 5(d) and 5(e)).

### 3.5. Heme Binding Attenuates the Proinflammatory Effect of Heme

**Figure 6:**
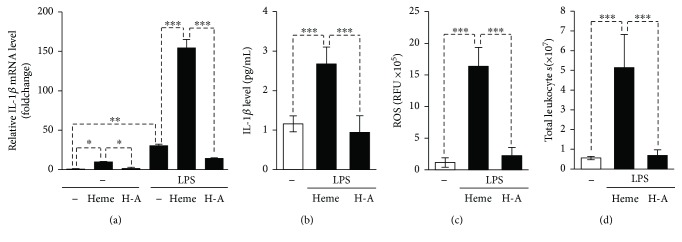
Albumin inhibits heme-induced NLRP3 inflammasome activation in HUVECs *in vitro* and heme-induced peritoneal infiltration of leukocytes *in vivo*. (a–c) HUVECs primed with LPS (10 *μ*g/mL, 24 h) were stimulated with heme or heme-albumin (H-A, 25 *μ*mol/L). (a) IL-1*β* mRNA level (4 h) was determined by quantitative RT-PCR. (b) Active IL-1*β* levels in cellular supernatants (24 h) were determined by ELISA. (c) ROS production (4 h) was measured with DCFDA assay. Results are shown as mean ± SD (*n* = 4) from one representative experiment of two. ^∗∗∗^*P* < 0.005, ^∗∗^*P* < 0.01, and ^∗^*P* < 0.05. (d) C57BL/6 mice were injected (i.p.) with heme (300 nmol/mice), heme-albumin (h–a, 300 nmol/mice), or PBS (*n* = 5 in all groups). Peritoneal cavity was rinsed, and the total number of leukocytes was counted after 16 h of treatment. Results are shown as mean ± SD, ^∗∗∗^*P* < 0.005.

Heme scavenging proteins such as hemopexin (Hx) or albumin block most of the prooxidant actions of heme [38]; therefore, we next investigated whether the proinflammatory actions of heme towards endothelial cells could be inhibited by albumin. We primed HUVECs with LPS and then challenged with heme or heme-albumin complex. In contrast to heme, heme-albumin failed to induce IL-1*β* mRNA expression and secretion of mature IL-1*β* in LPS-primed HUVECs (Figures 6(a) and 6(b)). In contrast to heme, heme-albumin did not increase ROS production in LPS-primed HUVECs (Figure 6(c)). Finally, we checked whether heme-albumin triggers leukocyte infiltration in C57BL/6 mice. Our results revealed that albumin completely inhibited heme-mediated leukocyte infiltration (Figure 6(d)). These results suggest that heme-binding plasma proteins exhibit an anti-inflammatory function in case of massive intravascular hemolysis by inhibiting heme-mediated inflammatory responses.

### 3.6. Oxidized Hb Forms Do Not Trigger Inflammasome Activation in Endothelial Cells

**Figure 7:**
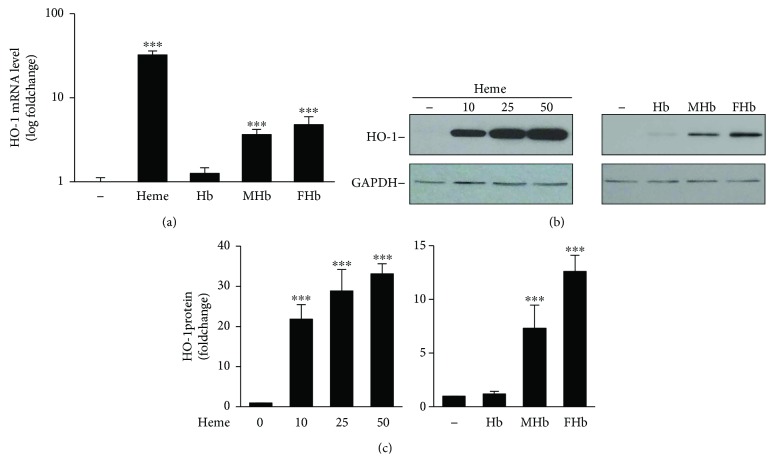
Oxidized Hb species transfer heme to endothelial cells. (a–c) HUVECs were treated with heme, Hb, metHb, or ferrylHb (25 *μ*mol/L heme group). (a) HO-1 mRNA level (4 h) was determined by quantitative RT-PCR. Results are shown as mean ± SD (*n* = 3) from one representative experiment of three. (b) Protein expression of HO-1 (8 h) was evaluated by Western blot. Membranes were reprobed for GAPDH. Representative blots of 3 independent experiments are shown. (c) Densitometric analysis of Western blots. Results are shown as mean ± SD of 3 independent experiments. ^∗∗∗^*P* < 0.005.

**Figure 8:**
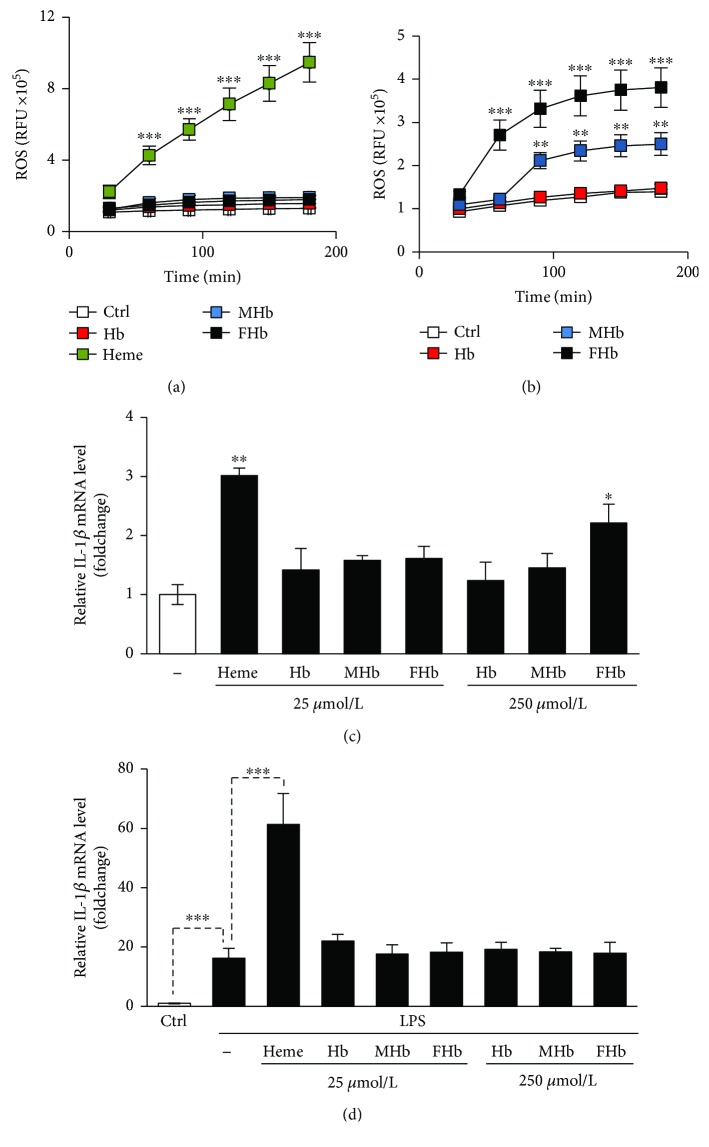
Oxidized Hb species increase ROS formation but fail to induce IL-1*β* production and maturation in HUVECs. (a–c) Naive (a–c) or LPS-primed (d) HUVECs were treated with heme (25 *μ*mol/L) Hb, metHb, or ferrylHb (25 or 250 *μ*mol/L). (a and b) Following the treatments (a: 25 *μ*mol/L and b: 250 *μ*mol/L) (4 h), ROS production was measured with DCFDA assay. (c and d) IL-1*β* mRNA level (4 h) was determined by quantitative RT-PCR. Results are shown as mean ± SD (*n* = 3) from one representative experiment of three. ^∗∗∗^*P* < 0.005, ^∗∗^*P* < 0.01, and ^∗^*P* < 0.05.

Oxidized forms of Hb are able to release their heme moiety; therefore, we next investigated whether the different Hb forms are involved in inflammasome activation and the subsequent production of IL-1*β* in HUVECs. Exposure of HUVECs to oxidized forms of Hb, that is, metHb and ferrylHb but not naive Hb resulted in the upregulation of the heme catabolizing enzyme heme oxygenase-1 (HO-1) in HUVECs as was revealed by quantitative RT-PCR and Western blotting (Figures 7(a) and 7(b)). Importantly, we found that metHb and ferrylHb are much weaker inducers of HO-1 than an equimolar amount of heme (Figures 7(a) and 7(b)). As heme-mediated ROS production is critical for NLRP3 inflammasome activation in HUVECs, we examined whether Hb species at different oxidation states increase intracellular ROS levels in HUVECs. In contrast to heme, none of the Hb forms at the concentration of 25 *μ*mol/L increased ROS production in HUVECs (Figure 8(a)). In contrast, when we applied the Hb forms at the concentration of 250 *μ*mol/L, we observed ROS production when the cells were treated with metHb and ferrylHb but not with naive Hb (Figure 8(b)).

Then, we investigated whether the different Hb forms induce NLRP3 inflammasome activation in HUVECs. First, we assessed IL-1*β* mRNA levels in nonprimed HUVECs treated with low (25 *μ*mol/L) or high (250 *μ*mol/L) concentration of Hb forms (Figure 8(c)). We found that high concentration of ferrylHb induced a 2.2-fold elevation of IL-1*β* mRNA expression in HUVECs, in which the effect was not observed in cells treated with metHb or naive Hb (Figure 8(c)). At the same time, heme at 10-times lower concentration caused an approximately 3-fold upregulation of IL-1*β* mRNA expression in HUVECs (Figure 8(c)). Finally, we investigated the effect of Hb forms (low and high doses) on IL-1*β* mRNA expressions in LPS-primed HUVECs. As shown in Figure 8(d), none of the Hb forms triggered further elevation of IL-1*β* mRNA levels when HUVECs were primed with LPS (Figure 8(d)). These results suggest that free heme, but not Hb-bound heme, is involved in inflammasome activation and the subsequent production of IL-1*β* in HUVECs.

## 4. Discussion

**Figure 9:**
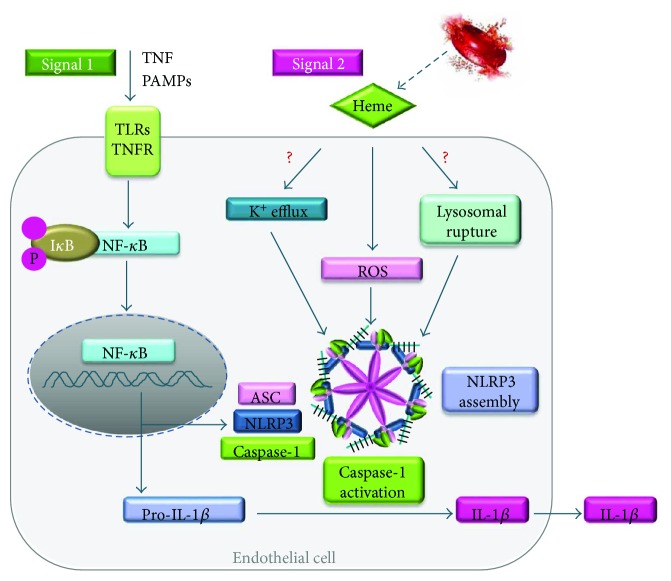
Working model of heme-induced NLRP3 inflammasome activation in endothelial cells. Pathogen-associated molecular patterns (PAMPs), such as LPS or TNF (Signal 1), bind to toll-like receptors (TLRs) or TNF receptor and prime endothelial cells to activate NF-*κ*B to induce the expression of NLRP3, caspase-1, and IL-1*β*. Heme (signal 2) that can derive from Hb released form damaged RBCs induces ROS generation, caspase-1 activation, and cleavage of pro-IL-1*β*. Mature form of IL-1*β* is secreted from the cell. The role of K^+^ efflux and lysosomal destabilization remained to be explored.

In this study, we show that heme is an inducer of IL-1*β* processing through the activation of the NLRP3 inflammasome in human endothelial cells. The molecular mechanism by which heme promotes NLRP3 activation involves ROS and requires structural integrity as well as “free”/non-Hb-bound status of heme (Figure 9).

Heme is a potent proinflammatory molecule in vivo, which is a notion supported by the finding that intraperitoneal injection of heme induces infiltration of neutrophils and monocytes/macrophages into the peritoneal cavity of mice. This inflammatory response is associated with increased production of the proinflammatory cytokine IL-1*β* in the peritoneum.

Under noninflammatory conditions, endothelial cells have multiple functions in maintaining blood fluidity, regulating blood flow, controlling vessel wall permeability, and keeping circulating leukocytes in a quiescent state. Upon infection or inflammation, endothelial cells are among the first cells coming into contact with microbial or endogenous molecules and they become active participants and regulators of the inflammatory response [23]. Endothelial cells are equipped with receptors of the innate immune system allowing them to sense and respond to a variety of pathogen-associated molecular patterns (PAMPs) and endogenous DAMPs [39].

Exposure of endothelial cells to classical DAMPs such as extracellular ATP and high mobility group box 1 protein (HMGB1) results in the activation of NLRP3 inflammasome and the subsequent production of IL-1*β* [40, 41]. Endothelial activation of NLRP3 inflammasome was observed in animal models of hypercholesterolemia and hyperglycemia, and endothelial production of IL-1*β* has been shown to contribute to diverse pathological conditions, including rheumatoid arthritis, hemorrhagic shock-induced acute lung injury, transfusion-related acute lung injury, and chronic kidney disease [27, 29, 32, 42–44].

Activation of different innate immune receptors including NLRP1, NLRP3, NLRC4, or AIM2 initiates the assembly of the inflammasome, leading to activation of inflammatory caspases and the maturation and secretion of IL-1*β* [45–48]. Previous studies showed that heme induces IL-1*β* production in macrophages through the activation of the NLRP3 inflammasome [21, 49]. Our results show that heme is a broader inducer of NLRP3 inflammasome activation and besides macrophages, its proinflammatory actions target endothelial cells as well. Activation of the NLRP3 inflammasome in macrophages requires two signals. The first (priming) signal provided mainly by toll-like receptors (TLRs) or TNF receptor 1 and 2, triggering NF-*κ*B-mediated expression of NLRP3 [50, 51]. The second signal is provided by a PAMP or DAMP that activates NLRP3 to trigger inflammasome assembly, activation of caspase-1, cleavage of pro-IL-1*β*, and release of the active cytokine [51]. In agreement with this notion, priming with LPS was shown to be essential for heme-mediated NLRP3 inflammasome activation in macrophages [21]. Regarding endothelial cells, heme induced low amount of active IL-1*β* formation in nonprimed HUVECs in which the response was largely amplified after LPS priming. Active IL-1*β* formation in nonprimed heme-treated HUVECs was associated with some degree of cell death. During endothelial cell necrosis, ATP and HMGB1 are released, which were shown to trigger endothelial NLRP3 inflammasome activation [40, 41, 52]. Therefore, it is possible that ATP and HMGB—mediators released upon heme-mediated cell death—contributed to heme-induced production of IL-1*β* in nonprimed HUVECs. On the other hand, LPS priming did not promote cell death but substantially increased the heme-mediated production of active IL-1*β*, suggesting that this response was independent of cell death.

Heme induced the expression of NLRP3 mRNA in HUVECs, regardless of LPS priming, and we showed that NLRP3 is an indispensable player in the heme-triggered production active IL-1*β*. As a result of NLRP3 inflammasome activation, the procaspase-1 zymogen is self-activated by proteolytic cleavage into the active form [51]. Activated caspase-1 then cleaves pro-IL-1*β* leading to the formation of the active cytokine [51]. We showed here that heme failed to induce the formation of activated caspase-1 and cleavage of pro-IL-1*β* in NLRP3 deficient mice.

Heme consists of a PPIX ring and a central Fe^2+^ ion that is stabilized by four N-Fe coordinate-covalent bonds. Following uptake by endothelial cells, heme is cleaved by HO-1 and the liberated d iron contributes to the labile iron pool of the cells. A recent study revealed that high intracellular iron in patients with sickle cell disease is associated with markers of inflammation and mortality [53]. Moreover, labile iron has been shown to induce NLRP3 inflammasome activation in human monocytes [54]. These observations inspired us to investigate whether free iron causes NLRP3 inflammasome activation in endothelial cells, too. Here, we show that in contrast to monocytes, iron itself is unable to trigger active IL-1*β* production in HUVECs. There are controversial results regarding NLRP3 inflammasome activation by the other component of heme, PPIX [21, 49]. Here, we show that PPIX fails to induce NLRP3 inflammasome activation and subsequent production of active IL-1*β* in LPS-primed endothelial cells. This is in agreement with the finding of Dutra et al. who showed that PPIX does not trigger active IL-1*β* production in LPS-primed macrophages [21]. Overall, our results suggest that coordinated iron present in the heme molecule is critical to the activation of NLRP3 inflammasome and subsequent production of active IL-1*β* in endothelial cells.

NLRP3 inflammasome activation is triggered by several structurally diverse molecules that share some common molecular mechanisms through which inflammasome activation occurs. These include elevated ROS production, K^+^ efflux, lysosomal damage, and ATP release [36, 55–57]. Because both heme and LPS are well-known inducers of ROS production in endothelial cells, here, we concentrated our work to investigate whether ROS is involved in heme-mediated NLRP3 inflammasome activation in LPS-primed HUVECs [18, 35]. Here, we show that LPS is a much weaker inducer of ROS production in HUVECs in comparison to heme. On the other hand, LPS priming increased heme-mediated ROS production in HUVECs in a synergistic manner, suggesting an interplay between the two triggers. Inhibition of ROS formation by NAC partially prevented heme-mediated production of IL-1*β* mRNA in LPS-primed HUVECs, suggesting that other mechanisms independently of ROS formation could contribute to this effect. Lysosomal destabilization has been shown to induce NLRP3 inflammasome activation in HUVECs [58, 59]. Previous work showed that certain activators of the NLRP3 inflammasome, such as bacterial pore-forming toxins and particulate matter, induce both mitochondrial ROS production and K^+^ efflux, but NLRP3 inflammasome activation was dependent exclusively on K^+^ efflux [55]. Dutra et al. showed that heme-mediated inflammasome activation is dependent on both ROS production and K^+^ efflux but independent on lysosomal destabilization in LPS-primed macrophages [21]. Further investigation is needed to see whether K^+^ efflux or lysosomal destabilization is involved in heme-mediated NLRP3 inflammasome activation in LPS-primed HUVECs and whether this effect is independent of ROS production.

Prooxidant and proinflammatory effects of circulating heme are controlled by specific and nonspecific heme-binding plasma proteins [18]. Hx is an acute-phase plasma protein that binds heme with the highest affinity of any known protein and therefore it is the key defense against the deleterious effects of heme [38]. Along with this notion, Hx^−/−^ mice have increased renal damage after acute hemolysis in comparison to wild-type mice [60]. Exogenous administration of Hx protects mice against endothelial damage triggered by heme overload by improving liver and cardiovascular functions [61, 62]. Besides Hx, other plasma proteins exhibit heme-binding activity that can support the defense system upon massive hemolysis when Hx is depleted [18]. For example, the abundant plasma protein albumin has one strong binding site for heme (fatty acid binding site and FA1 domain) [63]. Here, we showed that albumin binding inhibits heme-mediated NLRP3 inflammasome activation in LPS-primed HUVECs *in vitro* and blocks heme-mediated peritoneal leukocyte infiltration *in vivo* in C57BL/6 mice. In accordance with our finding, Dutra et al. showed previously that Hx binding inhibits heme-mediated NLRP3 inflammasome activation in LPS-primed macrophages [21].

Oxidation of Hb in the extracellular milieu is an event of crucial interest in pathological hemolytic conditions, because only oxidized forms of Hb are able to release their heme moiety. In line with this notion, metHb and ferrylHb, similarly to that of free heme, sensitize endothelial cells to oxidant-mediated killing but naive Hb lacks such harmful effect [22, 64]. Additionally, ferrylHb possesses proinflammatory actions towards endothelial cells leading to endothelial cell activation and the disruption of endothelial barrier function, in which the actions are independent of heme release and are exclusively linked to ferrylHb as neither Hb nor metHb behave in a proinflammatory manner towards endothelial cells [25, 65]. Endothelial cells exposed to heme upregulate HO-1, the rate-limiting enzyme of heme degradation. Here, we confirmed that oxidized forms of Hb, that is, metHb and ferrylHb release their heme moiety by assessing HO-1 mRNA and protein expressions in HUVECs following exposure of different Hb forms. Our data revealed that metHb and ferrylHb are much weaker inducers of HO-1 than free heme, suggesting that heme release from oxidized Hb forms is not complete. This is in agreement with previously reported studies in which the ability of different Hb forms in triggering oxidative modification of low-density lipoprotein (LDL) was examined [19, 64]. In comparing to heme, metHb and ferrylHb trigger a delayed and less pronounced oxidative modification of LDL, accompanying with lower numbers of LDL-associated heme groups, suggesting an incomplete release of heme moiety from oxidized Hb forms [19, 64]. Free heme induces ROS production in HUVECs, and we hypothesized that oxidized Hb forms accelerate ROS production in endothelial cells based on their ability to release heme. Indeed, we showed here that oxidized forms of Hb, namely, metHb and ferrylHb increase ROS production in HUVECs when applied at high concentrations. Our final question was whether oxidized Hb forms trigger NLRP3 inflammasome activation and subsequent production of IL-1*β* in HUVECs. Our data revealed that although high concentrations of metHb and ferrylHb increased ROS formation in HUVECs, they were not potent enough to significantly increase the level of IL-1*β* mRNA in HUVECs. Therefore, we concluded that in case of intravascular hemolysis, non-Hb-bound heme is the driving force of NLRP3 inflammasome activation in HUVECs.

## 5. Conclusion

In conclusion, we demonstrated that heme acts in a proinflammatory manner in vitro and in vivo and induces NLRP3 inflammasome activation and the subsequent production of the proinflammatory cytokine IL-1*β*. Besides macrophages, heme targets human endothelial cells and triggers the secretion of active IL-1*β*. Heme-mediated inflammatory response in HUVECs is largely amplified by LPS priming and was associated with unfettered ROS production. Heme triggers NLRP3 inflammasome activation only if it is structurally intact and if it is not bound to Hb or heme-binding proteins such as albumin. Further investigations are needed to explore whether other known mechanisms contributing to NLRP3 inflammasome activation such as K^+^ efflux or lysosomal destabilization are involved in heme-mediated NLRP3 inflammasome activation in HUVECs (Figure 9).

## Abbreviations

DAMPs: Damage-associated molecular patterns
FBS: Fetal bovine serum
ferrylHb, FHb: Ferryl hemoglobin
GAPDH: Glyceraldehyde 3-phosphate dehydrogenase
Hb: Hemoglobin
HBSS: Hank's balanced salt solution
HO-1: Heme oxygenase-1
HUVECs: Human umbilical vein endothelial cells
Hx: Hemopexin
ICAM-1: Intercellular adhesion molecule-1
IL-1*β*: Interleukin 1 beta
LDL: Low-density lipoprotein
LPS: Lipopolysaccharide
metHb, MHb: Methemoglobin
MTT: 3-[4,5-Dimethylthiazol-2-yl]-2,5-diphenyl-tetrazolium bromide
NAC: N-Acetyl cysteine
NLRP3: Nucleotide-binding domain leucine-rich repeat-containing protein 3
PAMPs: Pathogen-associated molecular patterns
PBS: Phosphate-buffered saline
PPIX: Protoporphyrin IX
RBCs: Red blood cells
TLR4: Toll-like receptor 4
TNF: Tumor necrosis factor
VCAM-1: Vascular cell adhesion molecule-1.
